# Strengthening Grapevine Resistance by *Pseudomonas fluorescens* PTA-CT2 Relies on Distinct Defense Pathways in Susceptible and Partially Resistant Genotypes to Downy Mildew and Gray Mold Diseases

**DOI:** 10.3389/fpls.2019.01112

**Published:** 2019-09-18

**Authors:** Sara Lakkis, Patricia Trotel-Aziz, Fanja Rabenoelina, Adrian Schwarzenberg, Eric Nguema-Ona, Christophe Clément, Aziz Aziz

**Affiliations:** ^1^Induced Resistance and Plant Bioprotection (RIBP), SFR Condorcet FR-CNRS 3417, University of Reims, UFR Sciences, Reims, France; ^2^Centre Mondial de l’Innovation, Groupe Roullier, Saint-Malo, France

**Keywords:** induced resistance, beneficial bacteria, *Pseudomonas fluorescens*, *Plasmopara viticola*, *Botrytis cinerea*, priming, *Vitis vinifera*

## Abstract

Downy mildew caused by the oomycete *Plasmopara viticola* and gray mold caused by the fungus *Botrytis cinerea* are among the highly threatening diseases in vineyards. The current strategy to control these diseases relies totally on the application of fungicides. The use of beneficial microbes is arising as a sustainable strategy in controlling various diseases. This can be achieved through the activation of the plants’ own immune system, known as induced systemic resistance (ISR). We previously showed that bacteria-mediated ISR in grapevine involves activation of both immune response and priming state upon *B. cinerea* challenge. However, the effectiveness of beneficial bacteria against the oomycete *P. viticola* remains unknown, and mechanisms underpinning ISR against pathogens with different lifestyles need to be deciphered. In this study, we focused on the capacity of *Pseudomonas fluorescens* PTA-CT2 to induce ISR in grapevine against *P. viticola* and *B. cinerea* by using two grafted cultivars differing in their susceptibility to downy mildew, Pinot noir as susceptible and Solaris as partially resistant. On the basis of their contrasting phenotypes, we explored mechanisms underlying ISR before and upon pathogen infection. Our results provide evidence that in the absence of pathogen infection, PTA-CT2 does not elicit any consistent change of basal defenses, while it affects hormonal status and enhances photosynthetic efficiency in both genotypes. PTA-CT2 also induces ISR against *P. viticola* and *B. cinerea* by priming common and distinct defensive pathways. After *P. viticola* challenge, PTA-CT2 primes salicylic acid (SA)- and hypersensitive response (HR)-related genes in Solaris, but SA and abscisic acid (ABA) accumulation in Pinot noir. However, ISR against *B. cinerea* was associated with potentiated ethylene signaling in Pinot noir, but with primed expression of jasmonic acid (JA)- and SA-responsive genes in Solaris, together with downregulation of HR-related gene and accumulation of ABA and phytoalexins.

## Introduction

Grapevine (*Vitis vinifera* L.) is a perennial crop species that is sensitive to a large spectrum of pathogens. Downy mildew, caused by *Plasmopara viticola* (Berk. & M. A. Curtis) Berl. & de Toni, and gray mold, caused by *Botrytis cinerea* (Pers.), are important diseases in many of the world’s viticultural areas. *P. viticola* is a biotrophic oomycete that attacks all green parts of the grapevine, while *B. cinerea* is a necrotrophic fungus that induces cell death to consume the host nutrients to spread (Dangl and Jones, 2001; Lenzi et al., 2016). The control of both diseases requires frequent fungicide applications to prevent significant crop losses. However, a growing concern about the impact of pesticides on the environment is motivating research for alternative strategies. The development of resistant cultivars is among the environmentally friendly alternatives to the use of chemicals. In this context, several resistance loci conferring resistance to *P. viticola* have been identified in *Muscadinia rotundifolia* (Bellin et al., 2009; Bouquet, 2011; Vezzulli et al., 2019), *Vitis riparia* (Marguerit et al., 2009), *Vitis amurensis* (Blasi et al., 2011; Schwander et al., 2012), and *Vitis cinerea* (Ochssner et al., 2016). However, *P. viticola* isolates are able to bypass or overcome resistance genes (Peressotti et al., 2010), thereby compromising the resistance sustainability in vineyards, while no source of resistance has been characterized against *B. cinerea* so far (Töpfer et al., 2011; Schwander et al., 2012).

Plant resistance can also be induced or strengthened after recognition of pathogenic or beneficial microbes through pathogen- or microbe-associated molecular patterns (PAMPs/MAMPs) by host-specific receptors. This resistance relies heavily on the ability of microorganisms to induce an immune signaling response known as PAMP-/MAMP-triggered immunity (PTI/MTI) (Jones and Dangl, 2006; Zipfel and Robatzek, 2010; Newman et al., 2013). Several beneficial bacteria, including *Pseudomonas* spp., have been shown to promote plant growth and to trigger activation of immune response at distant nonchallenged sites, thereby reducing plants’ susceptibility to pathogens, a process termed “induced systemic resistance” (ISR) (Verhagen et al., 2010; Berendsen et al., 2012; Pieterse et al., 2014; Gruau et al., 2015; Aziz et al., 2016). Evidence is accumulating that molecular processes associated with ISR are diversified and depend on the beneficial microorganism and the plant/pathogen system considered (Pieterse et al., 2014). In most cases, researches support the idea that ISR is characterized by priming the plant for a more efficient activation of various cellular defenses upon subsequent pathogen infection (Pozo et al., 2008; Van der Ent et al., 2009; Perazzolli et al., 2011). Priming can save plant energy costs and provides plants with an enhanced capacity for rapid and effective activation of cellular defense responses only once primed plants are attacked by a pathogen or an insect (Van Hulten et al., 2006; Conrath, 2011), whereby plants memorize previous microbial recognition and can respond more robustly to and counteract the subsequent invader attack.

The priming state involves distinct well-characterized responses, including upregulation of defense-related genes (Verhagen et al., 2004; Gruau et al., 2015), release of reactive oxygen species (Iriti and Faoro, 2003; Verhagen et al., 2010), callose deposition, and hypersensitive response (HR) (Trouvelot et al., 2008; Palmieri et al., 2012), as well as biosynthesis of antimicrobial phytoalexins (Yedidia et al., 2003; Aziz et al., 2016). Major defense phytohormones such as salicylic acid (SA), jasmonic acid (JA), ethylene (ET), and abscisic acid (ABA) take part in regulating the host immune response (Koornneef and Pieterse, 2008; Lopez et al., 2008; Bigeard et al., 2015). The effectiveness of phytohormones in induced resistance is mostly dependent on the pathogen’s infection strategy. The effective defense against (hemi)biotrophic pathogens was generally assumed to be dependent on the SA signaling pathway and HR-like cell death of the host, whereas the resistance against necrotrophic pathogens was largely associated to JA and ET pathways (Glazebrook, 2005; Pieterse et al., 2009). These generalities are controversial, especially in grapevine as JA signaling has been implicated in resistance against the biotrophic oomycete *P. viticola* (Figueiredo et al., 2015). This is in line with the fact that SA and JA signaling pathways are interdependent and most reports indicate a mutually antagonistic and also synergistic interactions between SA- and JA-dependent signaling (Pieterse et al., 2014). ABA has also an important functional role in defense responses, although its involvement in induced resistance to pathogen remains less obvious compared to the other phytohormones (Ton et al., 2009). Based on *Arabidopsis*, *Pseudomonas fluorescens*-mediated ISR functions independently of SA but requires a functional response to ET and JA without any increase in their production (Pieterse et al., 2000; Van der Ent et al., 2009; Pieterse et al., 2014). However, other *P. fluorescens* strains can induce ISR *via* SA signaling and not *via* JA/ET pathways (Van de Mortel et al., 2012). Other beneficial microbes including *Bacillus* sp. (Niu et al., 2011) and *Trichoderma* sp. (Palmieri et al., 2012) can also trigger ISR against hemibiotrophic and necrotrophic pathogens by inducing the expression of SA and ET/JA signaling pathways.

In grapevine, various beneficial bacteria have been identified regarding their effectiveness to prime plant immune response and to induce ISR against *B. cinerea* (Magnin-Robert et al., 2007; Trotel-Aziz et al., 2008; Verhagen et al., 2010,Verhagen et al., 2011; Gruau et al., 2015; Aziz et al., 2016; Miotto-Vilanova et al., 2016). However, to date, nothing is known about the efficacy of these bacteria against the oomycete *P. viticola*. Some nonpathogenic fungi including *Trichoderma harzianum* T39 (Perazzolli et al., 2008; Perazzolli et al., 2011; Perazzolli et al., 2012) and natural molecules such as β-aminobutyric acid (BABA) (Hamiduzzaman et al., 2005), chitosan (Aziz et al., 2006), and laminarin and its sulfated derivative (Aziz et al., 2003; Aziz et al., 2004; Trouvelot et al., 2008; Gauthier et al., 2014), as well as thiamine (Boubakri et al., 2012), have been reported to induce efficient resistance in grapevine against downy mildew. The T39- and BABA-induced resistance against *P. viticola* seems to be linked to primed JA-related signaling (Hamiduzzaman et al., 2005; Perazzolli et al., 2008), while laminarin and its sulfated form prime both JA- and SA-dependent pathways upon *P. viticola* challenge (Aziz et al., 2003; Trouvelot et al., 2008; Gauthier et al., 2014).

Research performed on *P. fluorescens* PTA-CT2 isolated from vineyard provided evidence that this bacterium is capable of colonizing grapevine roots but not the aboveground plant parts, inducing local and systemic immunity and boosting systemic resistance against *B. cinerea* (Trotel-Aziz et al., 2008; Verhagen et al., 2011; Gruau et al., 2015; Aziz et al., 2016). However, the molecular mechanisms governing this resistance have been only partially revealed, and the effectiveness of beneficial bacteria to control downy mildew caused by *P. viticola* remains to be deciphered. In this study, we focused on the ability of *P. fluorescens* PTA-CT2 to induce resistance in grapevine against *B. cinerea* and *P. viticola* by using two grafted grapevine cultivars differing in their susceptibility to downy mildew, Pinot noir as a sensitive cultivar and Solaris as a tolerant one, both grafted on the same rootstock under greenhouse conditions. Therefore, we investigated the capacity of grapevine plants to express basal and induced immune response at the systemic level upon interaction with *P. fluorescens* PTA-CT2, within a comparative context between susceptible and partially resistant cultivars. We especially evaluated the expression of defense-related genes by quantitative reverse-transcription PCR (RT-qPCR), including genes responsive to SA (*PR1*, *PR2*, and *GST1*) or involved in JA (*LOX9*) and ET biosynthetic (*ACO*) pathways, as well as HR-related (*HSR*) and phenylpropanoid-related genes (*PAL* and *STS*). Phytohormone and phytoalexin accumulations were also followed by liquid chromatography (LC)–tandem mass spectrometry (MS/MS) and ultra-performance LC (UPLC), and the consequence of the treatment on photosynthetic performance was evaluated by a pulse amplitude-modulated (PAM) fluorimeter. We also explored mechanisms underlying induced resistance triggered by PTA-CT2 upon challenge with the biotroph *P. viticola* and the necrotroph *B. cinerea* and addressed the similarities and differences in terms of phenotypic resistance and primed defense responses in both susceptible and partially resistant cultivars.

## Materials and Methods

### Plant Material and Growth Conditions

Two-year-old grapevine plants (*V. vinifera*), cultivar Pinot noir (PN114) as susceptible to *P. viticola* and Solaris 360 [Merzling × (Zaraya Severa × Muscat Ottonel)] as partially resistant and known to carry *Rpv3* and *Rpv10* loci, both grafted onto Kober 5BB rootstock, were individually planted in 4.5-L pots containing a compost–potting soil. Plants were grown in a greenhouse at a temperature of 24°C/day and 20°C/night, with 60% humidity and a photoperiod of 16-h light. Plants were watered twice a week. Three series of experiments were conducted over two consecutive years (2016 and 2017). After reaching the BBCH14 stage, plants were divided for each cultivar into three batches of five plants for the control and three batches of five plants for the bacterial treatment.

### Bacterial Treatment

*P. fluorescens* PTA-CT2 (GenBank Nucleotide Accession No. AM29367) was isolated from healthy filed-grown grapevine cv. Chardonnay in Champagne Area, France (Trotel-Aziz et al., 2008). Bacterial preculture was started by adding 10 µl of glycerol stock to 10 ml of sterile lysogeny broth (LB) medium and was incubated at 26°C with 110-rpm overnight shaking. This preculture was used for a new culture on the next day, following the protocol described in Gruau et al. (2015). Bacteria were then collected in their exponential growth phase (18 h of culture) by centrifugation at 5,000 × *g* for 10 min. Pellets were washed once and resuspended in 10 mM of sterile MgSO_4_. Concentration of the bacterial suspension was estimated by a spectrophotometer (450 and 650 nm) and was adjusted to 1 × 10^8^ colony-forming unit (CFU) ml^−1^ in sterile MgSO_4_. Three hundred milliliter of bacterial suspension was added at the root level of each pot, to reach a final concentration of 1 × 10^7^ CFU g^−1^ of soil. Bacterial suspension was applied once per year for 2016 and 2017. Control plants were similarly drenched with 300 ml of MgSO_4_. Both control and bacterium-treated plants were kept under greenhouse conditions mentioned above.

### Determination of Photosynthetic Efficiency

Chlorophyll fluorescence emission was measured with a PAM fluorimeter (Heinz Walz GmbH). Control and bacterium-treated plants were dark adapted for 10 min to determine the minimal level of fluorescence (Fo) and the maximal fluorescence (Fm) after a saturating pulse (0–20,000 µmol m^−2^ s^−1^). Leaves were then exposed to a halogen light source providing actinic light (0–3,000 µmol m^−2^ s^−1^). Photosynthetic parameters were measured on the uppermost third and fourth attached leaves (two leaves per plant). The Fv parameter indicates the maximum capacity for photochemical quenching. It is calculated by subtracting the Fo value from the Fm value. The ratio Fv/Fm reflects the maximum quantum efficiency of photosystem II (PSII), and ΦPSII indicates the photochemical yield of PSII. ETR corresponds to the electron transport rate of PSII and calculated according to the equation [ETR = *Y*(II) × PAR × 0.5 × PAR absorptivity] (Genty et al., 1989). Data are the means ± standard deviations from three independent experiments, each in triplicates, and each replicate consisted of five plants per cultivar over two consecutive seasons (2016 and 2017).

### Pathogen Growth Conditions, Infection, and Disease Evaluation

The *B. cinerea* strain 630 was grown on potato dextrose agar (PDA) medium and was incubated for 14 days at 22°C under continuous light for sporulation. Conidia were collected with 10 ml of sterile water as described in Aziz et al. (2003). The obtained suspension was filtered through sterile filter paper using a sterile syringe to remove the mycelium. Conidial concentration was determined by using a Malassez hemocytometer and adjusted with sterile water to 1 × 10^6^ conidia per milliliter.

The *P. viticola* isolate used in this study was purified as fresh single spore from infected leaves of the susceptible Pinot noir. Sporangia were propagated on *V. vinifera* cv. Pinot noir leaves in a glasshouse. To obtain sporangia, plants with oil spot symptoms were placed overnight in the dark at 100% relative humidity (RH). Inoculum of *P. viticola* sporangia was prepared by washing the freshly sporulating lesions with sterile distilled water. The sporangial suspension was then adjusted to a concentration of 10^5^ spores per milliliter using a Malassez hemocytometer.

Two weeks after the bacterial treatment, leaf disk assay was used for pathogen infection and disease evaluation. Leaf disks of 18-mm diameter were excised using the cork borer from the uppermost third and fourth leaves (two leaves per plant) of both control and bacterium-treated plants. The adaxial side of leaf disks was placed on a humidified Whatman paper in a Petri plate (12 disks per plate), and the abaxial side was inoculated with a 5-µl drop of a fresh suspension of *B. cinerea* (1 × 10^6^ conidia per milliliter) or 20-µl drop of a fresh suspension of *P. viticola* (1 × 10^5^ sporangia per milliliter). Inoculated leaf disks were placed in a growth chamber at 24°C with a 16- and 8-h photoperiod. Seven days after inoculation with *P. viticola*, all leaf disks were incubated overnight in the dark at 25°C with 95–100% RH.

Gray mold disease was evaluated at 7 days post infection (dpi) on 35–45 leaf disks from three independent experiments by scanning necrotic lesions caused by *B. cinerea* using Compu Eye, Leaf & Symptom Area (LSA) software (Bakr, 2005). Disease severity was also assessed at 7 dpi by scoring using a notation scale from 0 mm^2^ to more than 40 mm^2^ as follows: I, <5 mm^2^; II, between 5 and 10 mm^2^; III, between 10 and 20 mm^2^; IV, between 20 and 40 mm^2^; and V, >40 mm^2^ of necrosis area.

For *P. viticola*, disease intensity was also assessed at 8 dpi on 35–45 leaf disks from three independent experiments by measuring the leaf area covered by sporulation and necrotic lesions. The sporangial density of *P. viticola* was also determined. Sporangia were collected by suspending leaf disks in sterile distilled water and shaking for 1 h. Sporangia concentration was determined by direct counting with a Malassez hemocytometer under a light microscope. Disease rating was also expressed as the fraction of leaf disks falling in the following four classes for *P. viticola* infection: I, <5 mm^2^; II, between 5 and 10 mm^2^; III, between 10 and 20 mm^2^; and IV, >20 mm^2^. Data are the means ± standard deviations of the three experiments for both 2016 and 2017 seasons.

Gene expression, phytohormones, and phytoalexins were analyzed in both cultivars at 0 and 48 h after pathogen inoculation. The 48-h post infection (hpi) point was chosen as an optimal time for the high expression of most of the defense-related responses in both susceptible and partially resistant cultivars. To this end, the diameter of the leaf disks was reduced to 12 mm using a cork borer to eliminate the area where defense responses to injury occurred. Samples were immediately frozen in liquid N_2_ and stored at −80°C.

### RNA Extraction and RT-PCR

Leaf disks collected from control and bacteria-treated plants at 0 and 48 hpi were ground in liquid nitrogen. For each point, three biological replicates were used. Total RNA was extracted from 50 mg of leaf ground powder following the protocol of a PlantRNA purification reagent according to manufacturer instructions (Invitrogen, Pontoise, France). RNA pellet was suspended in 20 µl of RNase-free water and was incubated for 2 h at −20°C for solubilization. Genomic DNA was removed by DNase treatment, according to the manufacturer’s instruction (RQ1 RNase-free DNase, Promega). The concentration of the RNA was quantified using NanoDrop One by Thermo Fisher Scientific, and based on the obtained concentrations, all the samples were diluted to 100 ng µl^−1^. First-stranded complementary DNA (cDNA) was obtained from 100 ng of the total RNA using the Verso cDNA synthesis kit (Thermo Fisher Scientific).

The expression of eight genes known to be involved in defense was analyzed in both Pinot noir and Solaris plants. These genes encode glutathione-S-transferase (*GST1*), pathogenesis-related protein 1 (*PR1*), and β-1,3-glucanase (*PR2*) as markers of the SA pathway; 9-lipoxygenase (*LOX9*), an enzyme of the octadecanoid pathway; 1-aminocyclopropane carboxylic acid (ACC) oxidase (*ACO*) involved in ET biosynthesis; a HR-related gene (*HSR*) as a marker of cell death; phenylalanine ammonia-lyase (*PAL*), a key enzyme of the phenylpropanoid pathway; and stilbene synthase (*STS*), responsible for the biosynthesis of resveratrol. The primers for target genes were designed by Primer 3.0 software (Applied Biosystems) (Gruau et al., 2015) based on grapevine messenger RNA (mRNA) sequences deposited in GenBank. RT-PCR was performed with Absolute qPCR Mix, SYBR Green, ROX (Thermo Fisher Scientific), using a CFX96 system thermocycler (Bio-Rad). PCRs were carried out in 96-well plates to a final volume of 15 µl containing Absolute qPCR Mix, SYBR Green, ROX (*Taq* polymerase, dNTP, and SYBR Green Dye), 280 nM of forward and reverse primers (**Table S1**), and 30-fold diluted cDNA. Cycling parameters were as follows: 15 min of *Taq* polymerase activation at 95°C, followed by 40 two-step cycles composed of 10 s of denaturation at 95°C, and 45 s of annealing and elongation at 60°C. After the final cycle of the PCR, the specificity of each amplification was checked using melting analysis from 65°C to 95°C (**Figure S1**). Five commonly used reference genes, *EF1α* (elongation factor 1*α;* XM_002284888.3), *60SRP* (60S ribosomal protein L18; XM_002270599.3), *ADH2* (alcohol dehydrogenase; NM_001281154.1), *39SRP* (39S ribosomal protein L41-A; XM_002285709.4), and *UBE2* (ubiquitin-conjugating enzyme E2C; XM_002275879.4) (Gruau et al., 2015), were evaluated with Bio-Rad CFX Manager software v. 3.0 to select those with a stable expression in all tested conditions. *EF1* and *60SRP* genes were selected as the most stable reference genes in both grapevine cultivars and validated as internal controls for normalization for RT-qPCR analyses. PCR efficiency of primers was calculated by performing RT-PCR on serial dilutions of cDNA. All tested primer pairs were found to have amplification efficiencies of 90% to 103%.

Relative gene expression was determined with the formula fold induction: 2^−ΔΔCt^, using CFX Manager 3.0 software (Bio-Rad), where ΔΔCt = (Ct GI [unknown sample] − Ct GI [reference sample]) − (Ct GR [unknown sample] − Ct GR [reference sample]). GI is the gene of interest, and GR is the reference gene. Uninfected control of Pinot noir was considered as reference sample (1× expression level) for both cultivars. Integration of the formula for two reference genes was performed by the CFX Manager 3.0 software (Bio-Rad). The results presented correspond to the means of duplicate reactions of three independent biological replicates for the 2016 and 2017 seasons. The data were analyzed each year separately, since in 2016 the plants were treated once, while in 2017 the plants underwent a second treatment.

### Quantification of Phytohormones by LC–MS

Phytohormones were extracted from 20 mg of ground leaf powder in 1 ml of cold solution of methanol/water/formic acid (70/29/1, v/v/v). The homogenates were stirred at room temperature for 30 min and then centrifuged at 15,000 × *g* for 10 min at 4°C. The supernatant was evaporated under nitrogen, and the residue was dissolved with 1 ml of a 2% formic acid solution. The extracts were purified using a solid-phase extraction (SPE) EVOLUTE EXPRESS ABN 1 ml to 30 mg (Biotage, UK). The eluate was evaporated to dryness and dissolved in 200 µl of H_2_O containing 0.1% of formic acid.

Phytohormones, SA, JA, and ABA were purchased from OlChemIm (Olomouc, Czech Republic), and the precursor of ET, ACC, was purchased from Sigma-Aldrich (Saint-Quentin, France). Phytohormones were analyzed by an ultra-high-performance LC (UHPLC)–MS/MS system. Phytohormone analysis was achieved using a Nexera X2 UHPLC system (Shimadzu, Japan) coupled to a QTrap 6500+ mass spectrometer (SCIEX, Canada) equipped with an electrospray ionization (ESI) source. Two-microliter aliquots of purified extract were injected into a Kinetex EVO C18 core–shell column (100 × 2.1 mm, 2.6 µm, Phenomenex, USA) heated at 40°C. The mobile phases composed of Milli-Q water (solvent A) and acetonitrile LC–MS grade (Fisher Optima, UK) (solvent B), both containing 0.1% formic acid (LC–MS grade), were used to elute phytohormones with a flow rate of 0.7 ml min^−1^. The gradient elution started with 1% B, 0.0–5.0 min 60% B, 5.0–5.5 min 100% B, 5.5–7.0 min 100% B, 7.0–7.5 min 1% B, and 7.5–9.5 min 1% B. The ionization voltage was set to 5 kV for the positive mode and −4.5 kV for the negative mode, producing mainly [M+H]+ and [M−H]−, respectively. The analysis was performed in scheduled multiple-reaction monitoring (MRM) mode in positive and negative modes simultaneously with a polarity switching of 5 ms. Quantification was processed using MultiQuant software v. 3.0.2 (SCIEX, Canada). Data are the means ± standard deviations of the three independent experiments of the 2017 season.

### Phytoalexins Analysis by UPLC

Phytoalexins were extracted from 120 mg of freeze-dried powder of leaf disks with 2 ml of a solution of methanol/water (85/15: v/v) as described by Aziz et al. (2006). Samples were incubated on a shaker in the dark at room temperature for 2 h and then centrifuged for 10 min at 12,000 × g. The supernatants were collected in hemolysis tubes, and the remaining pellets were resuspended in 1 ml of 100% methanol and incubated for 1 h more on the shaker at room temperature and then centrifuged. The supernatants were pooled together and dried with a speed vacuum. The remaining residues were solubilized in 1 ml of pure methanol of LC–MS grade, filtered with 0.22-µm polytetrafluoroethylene (PTFE) filters into 2-ml amber vials. Phytoalexin analysis was performed using the ACQUITY™ UPLC system (Waters Corporation). Two-microliter aliquots were injected onto an ACQUITY UPLC BEH C18 1.7-µm, 2.1 × 100-mm column heated at 40°C. A mixture of water (A) and acetonitrile (B) containing 0.1% formic acid was used to elute phytoalexins with a flow rate of 0.5 ml min^−1^. Fluorescence was measured with an excitation wavelength of 330 nm and an emission wavelength of 375 nm using an ACQUITY fluorimeter (Waters). Signals were analyzed using Empower 2 software (Waters). Phytoalexins were quantified relative to retention time and calibration with external standards. Data are the means ± standard deviations of the three independent experiments for the 2016 season.

### Evaluation of the Direct Effect of PTA-CT2 on Pathogen Growth

Bioassays were performed on the leaves of intact plants of the susceptible cultivar Pinot noir. The abaxial leaf surface was sprayed with a suspension of PTA-CT2 at a concentration of 10^7^ CFU ml^−1^ using an atomizer, until homogenous coverage of the leaves was reached. Control leaves were sprayed with sterile water. Two hours later (a time supposed to be too early for the defense reaction to be active), leaf disks of 12-mm diameter were excised and placed with their adaxial side on a wetted filer paper and inoculated with 20 µl of *P. viticola* at 10^5^ sporangia per milliliter. Development of *P. viticola* was assessed on 20–24 leaf disks from three independent experiments at 7 dpi, and sporangia density was quantified using a Malassez hemocytometer under a light microscope.

*P. fluorescens* PTA-CT2 was also tested for its potential ability to inhibit *B. cinerea* growth *in vitro*. PTA-CT2 grown in LB medium was inoculated (5-µl drop at 10^7^ CFU ml^−1^) at four spots away from the center of a Petri plate containing PDA medium with a sterilized toothpick. Plates were then incubated at 28°C in the dark, and 24 h later, fresh *B. cinerea* mycelium was co-inoculated in the center of PDA plates. Control is PDA plates with a 5-µl LB drop. Plates were incubated at 24°C, and *B. cinerea* development was assessed 7 days later.

### Statistical Analysis

All experiments were repeated at least three times in both 2016 and 2017 seasons. For photosynthetic parameters, disease evaluation, direct effect, phytoalexins, and phytohormones, statistical analysis was performed using two-way analysis of variance (ANOVA) with the statistical program SPSS 20 software using *post hoc* Tukey’s test honestly significant difference (HSD) to detect significant difference (*P* < 0.05) between the treatments. For RT-qPCR experiments, transcripts differentially expressed between control and bacteria-treated plants before and after pathogen infection in Pinot noir and Solaris were analyzed by comparing the expression means of duplicate reactions of three independent experiments in each treatment and cultivar over the 2016 and 2017 seasons.

## Results

### In the Absence of Pathogen Infection, PTA-CT2 Does Not Elicit Any Consistent Change of Basal Defenses

**Figure 1:**
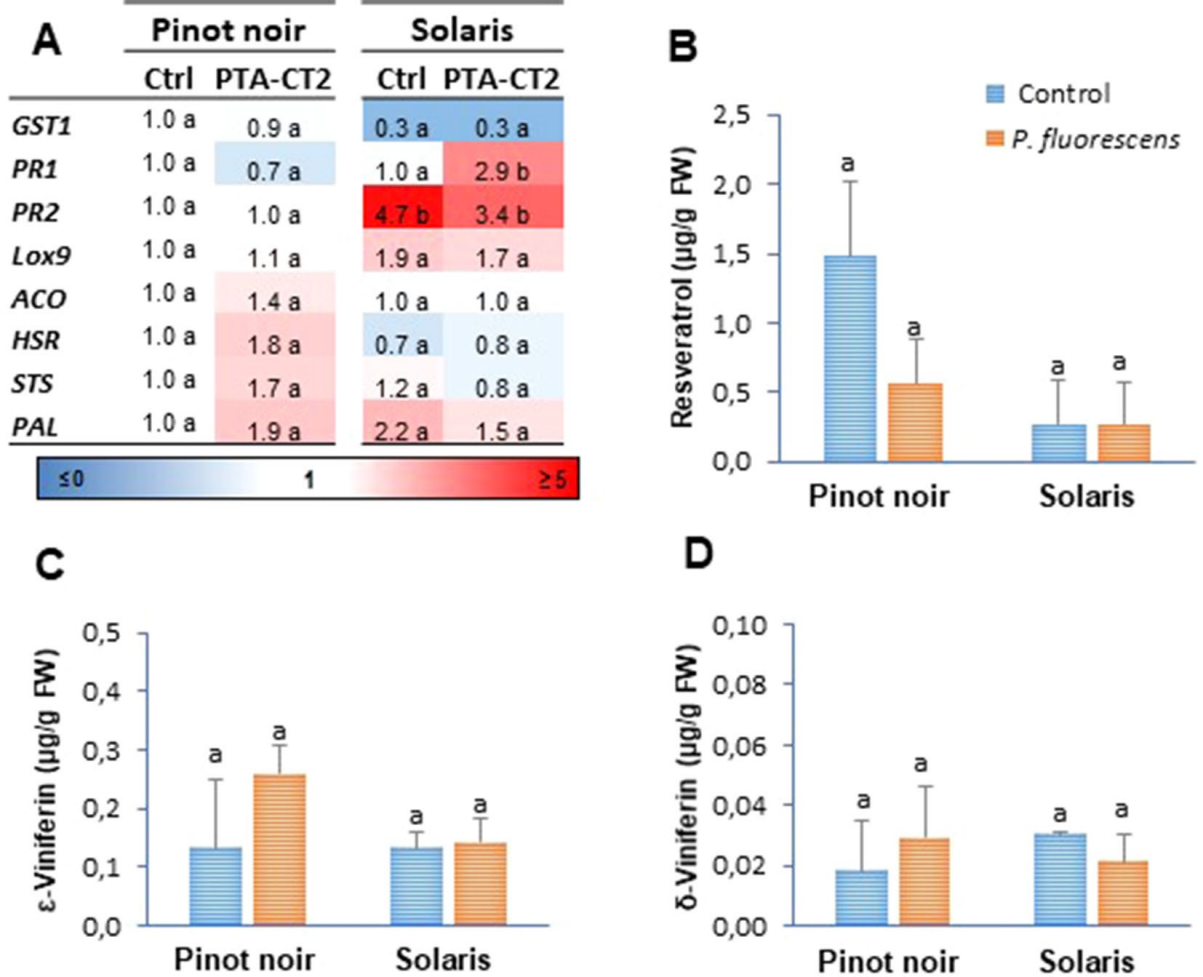
*Pseudomonas fluorescens* PTA-CT2 has no effect on the basal expression of defense-related genes and phytoalexin production in the leaves of grapevine cultivars Pinot noir and Solaris. Plants from each cultivar were treated at the root level with *P. fluorescens* at 10^7^ CFU g^−1^ of soil, and 2 weeks later, the transcript level of defense genes **(A)** and the amounts of resveratrol **(B)**, *ε*-viniferin **(C)**, and *δ*-viniferin **(D)** were quantified. For gene expression, uninfected control of Pinot noir was considered as the reference sample (1× expression level) for both cultivars, and heatmaps represent changes in transcript expression levels as indicated by the color shading. *GST1*, glutathione S-transferase 1; *PR1*, pathogenesis-related protein 1; *PR2*, *β*-1,3-glucanase; *LOX9*, lipoxygenase 9; *ACO*, 1-aminocyclopropane-1-carboxylic acid oxidase; *HSR*, hypersensitive related; *PAL*, phenylalanine ammonia-lyase; *STS*, stilbene synthase. Data are the means of three independent experiments performed in 2016, and vertical bars indicate standard deviation. Different letters indicate statistically significant differences between the treatments (ANOVA Tukey test, *P* < 0.05).

In this study, we evaluated by RT-qPCR the expression of eight selected defense genes from the different functional categories: genes encoding a glutathione-S-transferase (*GST1*), a pathogenesis-related protein 1 (*PR1*), and a *β*-1,3-glucanase (*PR2*) as markers of the SA signaling pathway; a 9-lipoxygenase (*LOX9*) involved in oxylipin synthesis as a marker of the JA pathway; a ACC oxidase (*ACO*) that catalyzes the conversion of ACC to ET; a HR-related (*HSR*) gene as a marker of cell death; a phenylalanine ammonia-lyase (*PAL*) catalyzing the first step in the phenylpropanoid pathway; and a stilbene synthase (*STS*) encoding a key enzyme of the resveratrol (a grapevine phytoalexin) biosynthesis. Data (**Figure 1A**) showed certain differences in the level of basal PR gene expression between the two grafted grapevine cultivars under greenhouse conditions. The partially resistant Solaris exhibited higher constitutive expression of PR2 transcripts compared to the susceptible Pinot noir. However, the application of PTA-CT2 at the root level does not induce any consistent change in gene expression at the systemic level in the absence of pathogen infection (**Figure 1A**). For most transcripts, the fold change ratio in PTA-CT2- over mock-treated plants was close to the corresponding control in both cultivars, except PR1, which was upregulated in Solaris.

Given the role of phytoalexins in plant defense and their known antimicrobial activity, we also investigated stilbene concentration in grapevine leaves after root treatment with PTA-CT2. In both cultivars, PTA-CT2 did not cause any significant production of resveratrol (3,5,4′-trihydroxystilbene) (**Figure 1B**) or its dimers *?*-viniferin (**Figure 1C**) and δ-viniferin (**Figure 1D**). The basal level of these stilbenes remained also comparable between the two grape varieties.

### PTA-CT2 Induces Differential Production of Phytohormones in Susceptible and Resistant Cultivars

**Figure 2:**
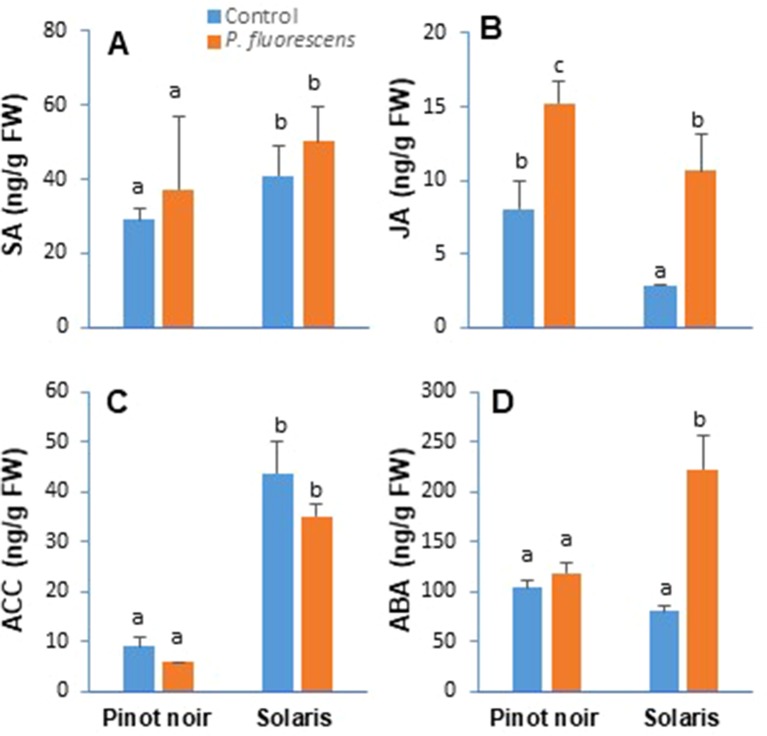
*Pseudomonas fluorescens* PTA-CT2 induces differential production of phytohormones in the leaves of grapevine cultivars Pinot noir and Solaris. Plants were treated at the root level with *P. fluorescens* at 10^7^ CFU g^−1^ of soil, and 2 weeks later, the amounts of salicylic acid **(A)**, jasmonic acid **(B)**, 1-aminocyclopropane-1-carboxylic acid **(C)**, and abscisic acid **(D)** were quantified by liquid chromatography (LC)–tandem mass spectrometry (MS/MS). Data are the means from three independent experiments performed in 2017, and vertical bars indicate standard deviation. Different letters indicate statistically significant differences between the treatments (ANOVA Tukey test, *P* < 0.05).

Plant hormones have a critical role as cellular signaling molecules with a central function in the regulation of immune response. To assess whether PTA-CT2 impacts systemic changes in phytohormone production, the levels of SA, JA, ACC (ET precursor), and ABA were determined by LC–MS/MS in the leaves of both Pinot noir and Solaris upon treatment of the roots with PTA-CT2. Data (**Figure 2**) showed significant cdifferences in the level of phytohormones between cultivars. Higher levels of SA (**Figure 2A**) and ACC (**Figure 2C**) were recorded in the leaves of nontreated Solaris compared to nontreated Pinot noir, which produced more JA (**Figure 2B**). However, any difference was observed between nontreated grape varieties regarding the level of ABA in the leaf tissues (**Figure 2D**). Upon treatment of the roots with PTA-CT2 bacteria, neither the SA content (**Figure 2A**) nor the level of ACC (**Figure 2C**) was altered in the leaves of both cultivars. However, a significant increase of JA level (**Figure 2B**) was observed in PTA-CT2-treated plants compared to their respective controls. The highest level of JA was recorded in the susceptible cultivar. The production of ABA in the leaves did not change in Pinot noir but strongly increased in Solaris after roots’ treatment with PTA-CT2. These results indicate that grape cultivars displayed contrasting levels of phytohormones and that the bacterium does not impact SA and ACC levels, which were highly produced in the downy mildew-resistant Solaris. However, the production of JA and ABA was differentially increased by PTA-CT2 in grapevine cultivars.

### PTA-CT2 Enhances Photosynthetic Capacity in Both Susceptible and Resistant Cultivars

**Figure 3:**
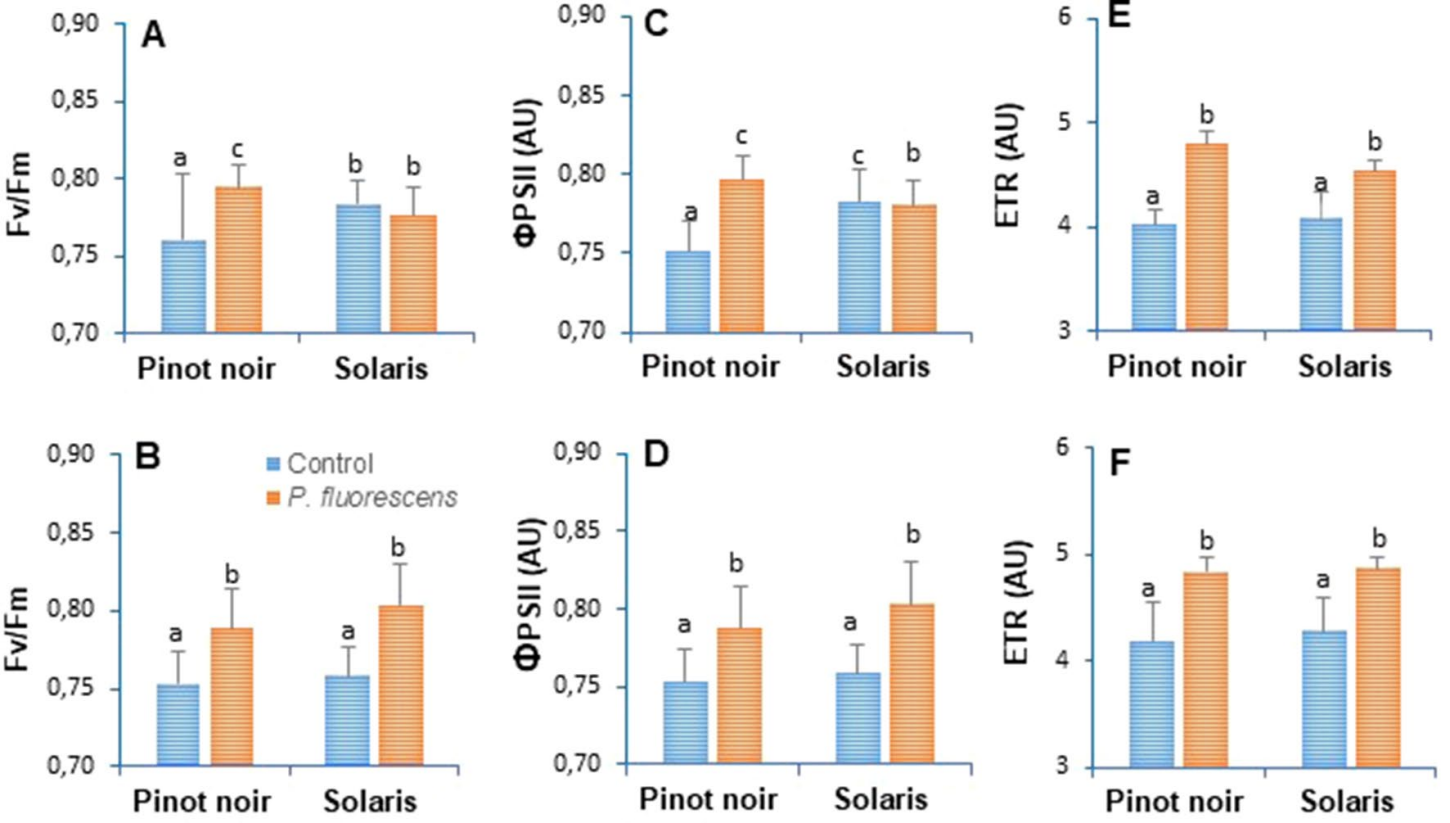
*Pseudomonas fluorescens* PTA-CT2 stimulates photosynthetic efficiency in both grapevine cultivars Pinot noir and Solaris. Plants were treated at the root level with *P. fluorescens* at 10^7^ CFU g^−1^ of soil, and 2 weeks later, the maximum photochemical efficiency of the photosystem II (PSII) (Fv/Fm, **A** and **B**), photochemical yield of PSII (*Φ*PSII, **C** and **D**), and relative photosynthetic electron transport (ETR, **E** and **F**) were determined by pulse amplitude-modulated (PAM) fluorimeter. Data expressed in arbitrary units (AU) of chlorophyll fluorescence are the means from three independent experiments performed in 2016 **(A, C, E)** and 2017 **(B, D, F)**, and vertical bars indicate standard deviation. Different letters indicate statistically significant differences between the treatments (ANOVA Tukey test, *P* < 0.05).

Activation of plants defense responses is often accompanied by an increased energy supply deriving from photosynthesis (Shoresh et al., 2010). In order to evaluate the effect of PTA-CT2 on physiological status, photosynthetic parameters such as maximum photochemical efficiency of PSII (Fv/Fm), photochemical yield of PSII (ΦPSII), and relative photosynthetic electron transport (ETR) were measured on attached leaves from control and treated plants (**Figure 2**). Data revealed significant differences between controls of Pinot noir and Solaris in terms of the photosynthetic efficiency of PSII, especially in 2016 when Fv/Fm (**Figure 3A**) and ΦPSII (**Figure 3C**) were higher in Solaris than in Pinot noir. Clear differences were also recorded between PTA-CT2- and mock-treated plants in both Pinot noir and Solaris genotypes. PTA-CT2-treated plants showed a significant increase of photosynthetic efficiency as indicated by elevated values of Fv/Fm (**Figures 3A, B**), ΦPSII (**Figures 3C, D**), and ETR rate (**Figures 3E, F**) in both cultivars, highlighting an improvement of energy transfer within PSII after root treatment with PTA-CT2.

### PTA-CT2 Induces ISR Against *P. viticola* and *B. cinerea* to Different Extents in Susceptible and Resistant Cultivars

**Figure 4:**
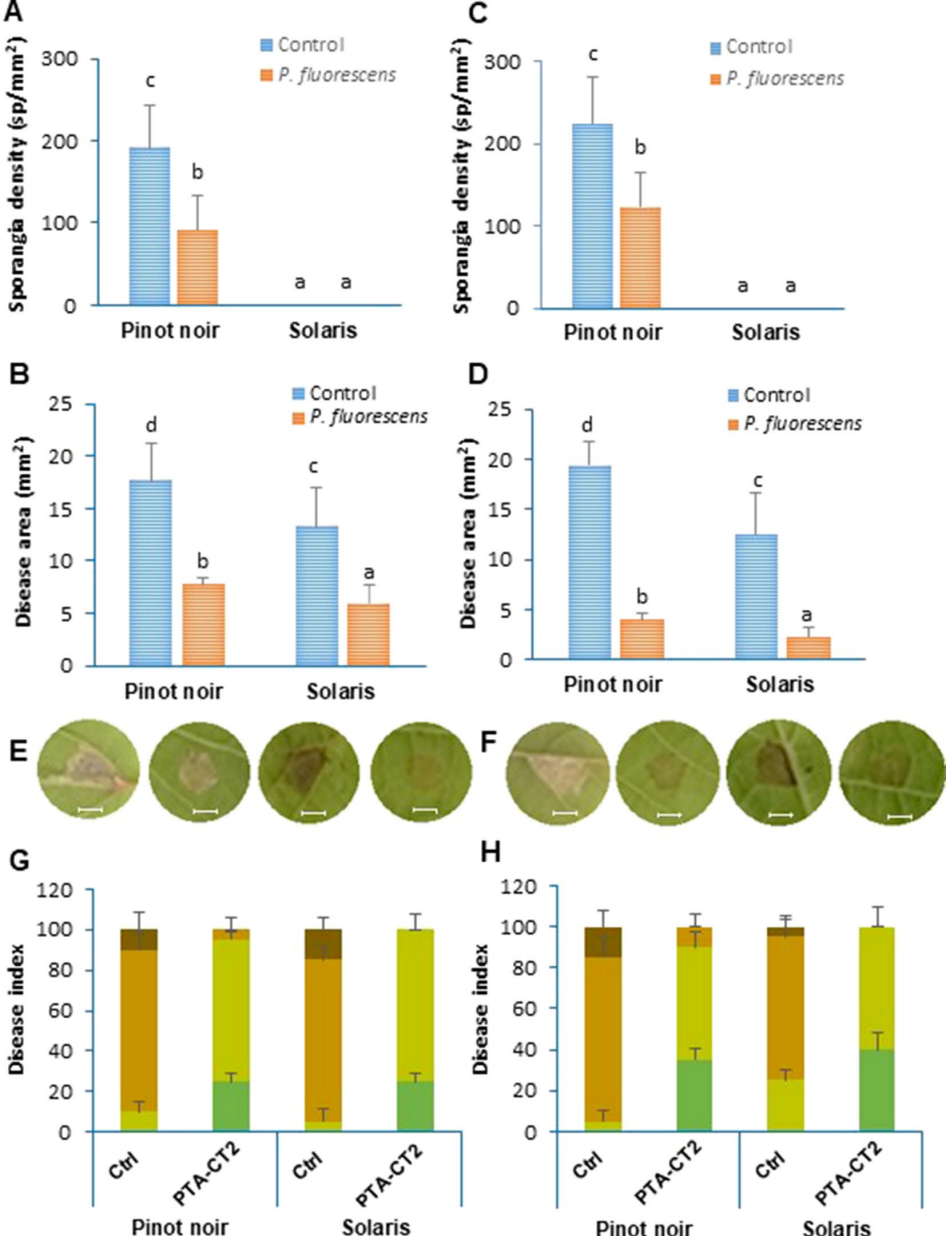
*Pseudomonas fluorescens* PTA-CT2 strengthens systemic resistance against *Plasmopara viticola* in both susceptible cultivar Pinot noir and partially resistant cultivar Solaris. Plants were treated at the root level with *P. fluorescens* at 10^7^ CFU g^−1^ soil, and 2 weeks later, leaf disks were excised from the upper third and fourth leaves and infected with 20 µl of 10^5^ spores per milliliter of *P. viticola*. Sporangia density was quantified using Malassez hemocytometer at 7 days post infection (dpi) **(A, B)**, and necrotic lesions covered or not with spores were evaluated 7 dpi by Compu Eye, Leaf & Symptom Area (LSA) software **(C, D)**. Photographs **(E, F)** depict the representative disease symptoms on leaf disks of control and PTA-CT2-treated plants at 7 dpi. Bars = 4 mm. Disease index **(G, H)** was expressed as the fraction of leaf disks falling in the following classes: dark green, no visible symptom; light green, lesion covering 5–10 mm^2^; light brown, lesion covering 10–20 mm^2^; dark brown, lesion covering more than 20 mm^2^ of the leaf disk area. Data are the means from three independent experiments, each experiment was performed with 30 leaves for each condition in 2016 **(A, C, E, G)** and 2017 **(B, D, F, H)**, and vertical bars indicate standard deviation. Different letters indicate statistically significant differences between the treatments (ANOVA Tukey test, *P* < 0.05).

The capacity of beneficial bacteria to trigger ISR in the susceptible cultivar Pinot noir and the partially resistant Solaris was assessed by drenching the soil with *P. fluorescens* PTA-CT2 at a concentration of 10^7^ CFU g^−1^ of soil. Two weeks later, leaf disks were excised from control and treated plants and infected with *P. viticola* and *B. cinerea*. Disease symptoms were monitored 7 dpi by quantifying sporulation of *P. viticola* over the seasons of 2016 and 2017 (**Figures 4A, B**) and scanning necrotic lesions covered or not with spores (**Figures 4C, D**). Inoculated leaf disks were also ranked into four classes according to the severity of symptoms (**Figures 4G, H**). As expected, leaf disks of control Pinot noir were heavily colonized by spores of *P. viticola*, while no sporulation was observed in the partially resistant cultivar Solaris (**Figures 4A, B**). However, necrotic spots can be seen in the Solaris cultivar after 7 days of infection (**Figures 4E, F**). Based on sporulation symptoms in the susceptible cultivar Pinot noir, we can see that PTA-CT2 reduced sporulation density of *P. viticola* by about 54%. Based on necrotic lesions together with area covered with spores, data provide evidences that the lesion sizes in nontreated Pinot noir were larger than those in nontreated Solaris over the consecutive years (**Figures 4C, D**). Treatment with PTA-CT2 reduced significantly the disease severity caused by *P. viticola* in both 2016 and 2017. PTA-CT2 reduced development of *P. viticola* in the susceptible cultivar Pinot noir by about 80%, while in the partially resistant cultivar Solaris, necrotic lesions caused by *P. viticola* were reduced by about 55%. This protection seems to be more pronounced in both cultivars, which underwent a second treatment in 2017 (**Figure 4D**).

The difference between genotypes was also clear according to the severity of disease symptoms (**Figures 4G, H**). The number of leaf disks that displayed extensive lesions was much higher in the susceptible cultivar Pinot noir than in the partially resistant cultivar Solaris. In 2017 (**Figure 4H**), the disease rating caused by *P. viticola* in Pinot noir was higher, with approximately 92% of leaf disks heavily infected compared (**Figure 4G**) to 40% in Solaris (**Figure 4H**). However, the severity of the disease was strongly reduced in PTA-CT2-treated plants of both cultivars compared to the infected control in 2016. According to disease scoring in 2017, PTA-CT2 also reduced downy mildew severity by approximately 40% in both varieties. These results indicate that PTA-CT2 can induce resistance against *P. viticola* under greenhouse conditions by severely limiting downy mildew symptoms in leaf tissues.

**Figure 5:**
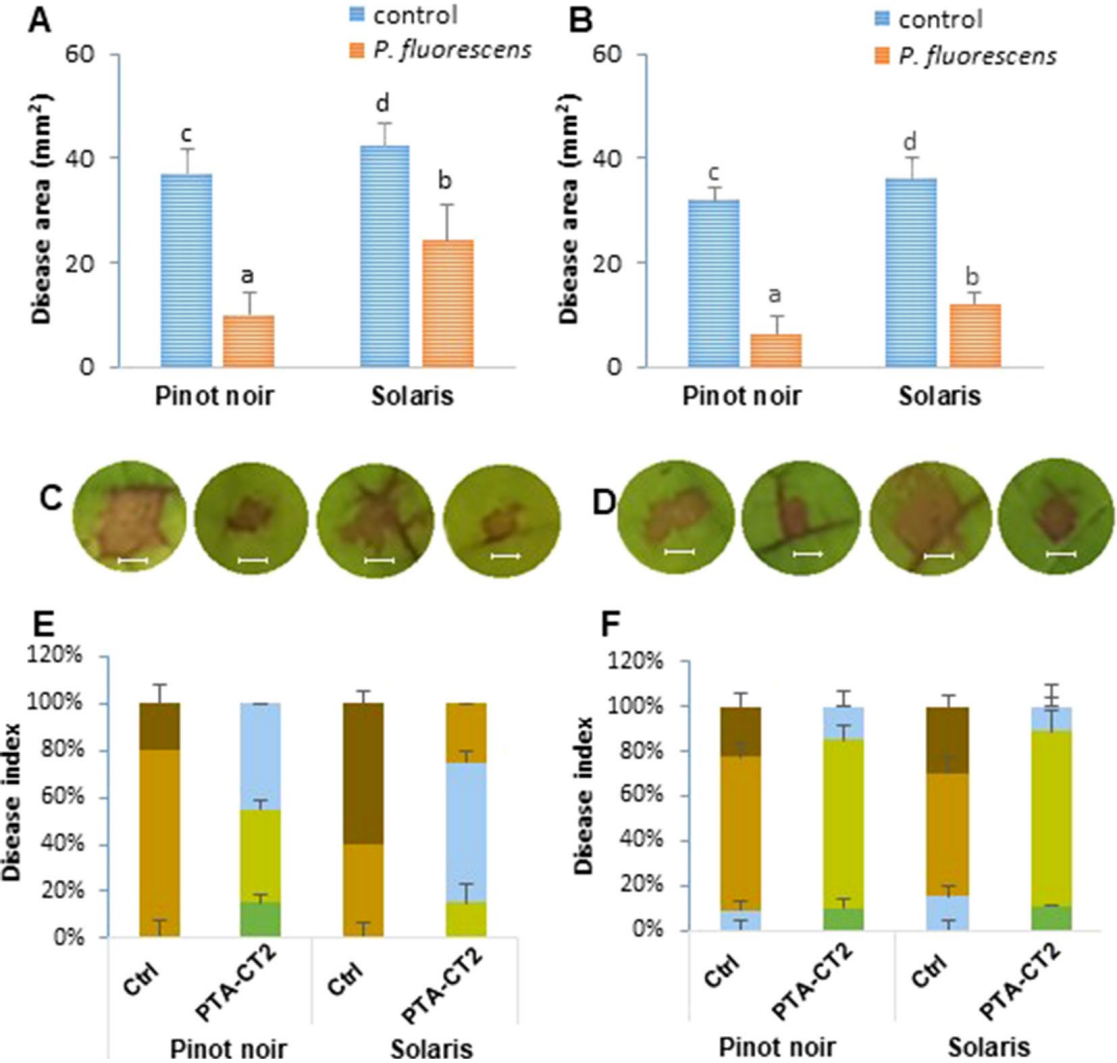
*Pseudomonas fluorescens* PTA-CT2 induces systemic resistance against *Botrytis cinerea* in Pinot noir and Solaris cultivars. Plants were treated at the root level with *P. fluorescens* at 10^7^ CFU g^−1^ of soil, and 2 weeks later, leaf disks were excised from the upper third and fourth leaves and infected with 5 µl of 10^6^ conidia per milliliter of *B. cinerea*. Necrotic lesion sizes were evaluated at 7 dpi by Compu Eye, Leaf & Symptom Area (LSA) software **(A, B)**. Photographs **(C, D)** depict the representative disease symptoms on leaf disks of control and PTA-CT2-treated plants at 7 dpi. Bars = 4 mm. Disease index **(E, F)** was expressed as the fraction of leaf disks falling in the following classes: dark green, no visible symptom; light green, lesion covering 5–10 mm^2^; blue, lesion covering 10–20 mm^2^; light brown, lesion covering 20–40 mm^2^; dark brown, lesion covering more than 40 mm^2^ of the leaf disk area. Data are the means from three independent experiments, each experiment was performed with 30 leaves for each condition in 2016 **(A, C, E)** and 2017 **(B, D, F)**, and vertical bars indicate standard deviation. Different letters indicate statistically significant differences between the treatments (ANOVA Tukey test, P < 0.05).

Pinot noir and Solaris displayed also contrasting susceptibility phenotypes to *B. cinerea* (**Figure 5**). Over the two consecutive years, the nontreated Solaris showed very large necrosis caused by *B. cinerea* than the nontreated Pinot noir (**Figures 5A–D**). The difference of phenotypic susceptibility to *B. cinerea* was also apparent on a large number of leaf disks according to the disease symptoms (**Figures 5E, F**). However, in both cultivars, a limited progression of gray mold disease was observed after treatment with PTA-CT2. This bacterium appeared more efficient in Pinot noir than in Solaris, since the disease area was reduced by about 73% to 80% in Pinot noir over 2016 (**Figure 5A**) and 2017 (**Figure 5B**), respectively, while in Solaris the disease was reduced by 43% (**Figure 5A**) to 66% (**Figure 5B**). Similarly, according to the severity of disease symptoms of *B. cinerea* (**Figures 5E, F**), the number of leaf disks that displayed extensive lesions was much higher in nontreated Solaris than in nontreated Pinot noir. This phenotypic difference was especially apparent in 2016 than in 2017 year. Following bacterial treatment, inoculated leaf disks with *B. cinerea* showed weaker symptoms than the control in both cultivars (**Figures 5E, F**). The number of leaf disks with extensive spreading lesions was strongly reduced in the PTA-CT2-treated plants. Most of the leaf disks exhibited weak spreading lesions, with 5% to 10% disease area in Pinot noir and 10% to 20% in Solaris in 2016 (**Figure 5E**). This marked reduction of gray mold symptoms was similar in both cultivars in 2017 with a large proportion of leaf disks being weakly or not infected. This suggests that the mechanisms involved in mediated protection against *P. viticola* may differ from those involved against *B. cinerea*.

The direct effects of PTA-CT2 on *P. viticola* and *B. cinerea* were also evaluated in co-inoculation assays using intact leaves from Pinot noir and PDA medium, respectively. In both cases, we did not record any inhibitory effect of the bacterium compared to the control. The sporulation of *P. viticola* and mycelial development of *B. cinerea* were similar to that of the untreated control (**Figure S2**). Therefore, given the spatial separation of challenging pathogens and the inducing bacterium, it can be inferred that the mediated protection against downy mildew and gray mold diseases resulted from an induced ISR in grapevine plants. We thus took advantage of the contrasting susceptibility and induced resistance phenotypes of both cultivars to explore mechanisms underpinning ISR against pathogens with different lifestyles.

### PTA-CT2 Primes Differential Defensive Pathways Against *P. viticola* and *B. cinerea* in Susceptible and Partially Resistant Cultivars

**Figure 6:**
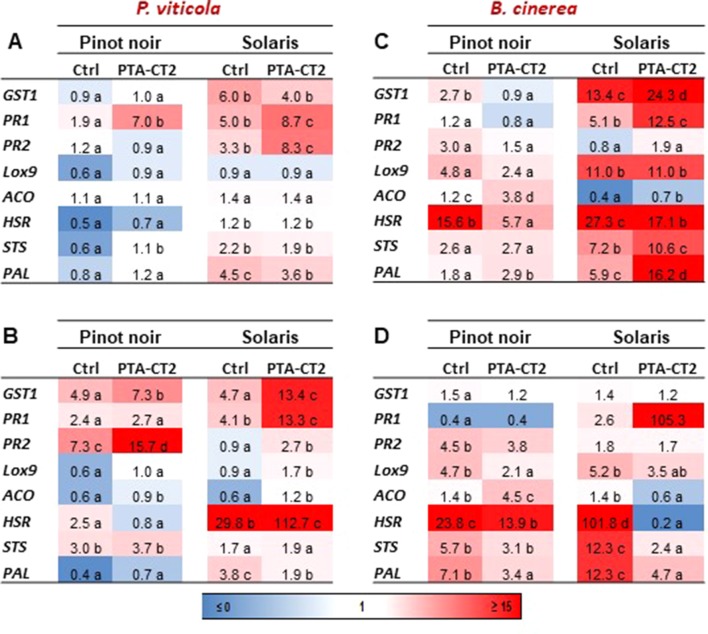
*Pseudomonas fluorescens* PTA-CT2 primes differential expression of defense-related genes in leaf disks of Pinot noir and Solaris after *Plasmopara viticola* and *Botrytis cinerea* inoculation. Grafted plants were treated with *P. fluorescens* PTA-CT2 at the root level, and 2 weeks later, leaf disks were excised from control and treated plants and infected with 20 µl of 10^5^ spores per milliliter of *P. viticola* **(A, B)** or 5 µl of 10^6^ conidia per milliliter of *B. cinerea* **(C, D)**. The transcript level of defense-related genes was determined by quantitative reverse-transcription PCR (RT-qPCR) at 2 days post inoculation. Uninfected control of Pinot noir was considered as a reference sample (1× expression level) for both cultivars, and heatmaps represent changes in transcript expression levels as indicated by the color shading. Data are the means from three independent experiments performed in 2016 **(A, C)** and 2017 **(B, D)**. Different letters indicate statistically significant differences between the treatments (ANOVA Tukey test, *P* < 0.05). *GST1*, glutathione-S-transferase 1; *PR1*, pathogenesis-related protein 1; *PR2*, *β*-1,3-glucanase; *LOX9*, lipoxygenase 9; *ACO*, 1-aminocyclopropane-1-carboxylic acid oxidase; *HSR*, hypersensitive related; *PAL*, phenylalanine ammonia-lyase; *STS*, stilbene synthase.

To investigate the capacity of *P. fluorescens* PTA-CT2 to prime immune defense at the systemic level, the expression of defense-related genes responsive to different signaling pathways was monitored in both cultivars at 48 hpi with the biotrophic oomycete *P. viticola* and the necrotrophic fungus *B. cinerea*. As shown before, in the absence of pathogen infection, PTA-CT2 did not affect basal gene expression in either cultivar. This indicates that the onset of ISR is not associated with the change of defense responses before pathogen challenge but may involve the priming state of plants for more rapid and strong activation of immune defense upon pathogen inoculation. The relative expression of defense genes was then compared between *P. viticola*-, *B. cinerea*-, and mock-inoculated leaf disks of both Pinot noir and Solaris after pretreatment with PTA-CT2. As seen in **Figure 6**, the expression of targeted genes showed differential responsiveness between cultivars after challenge. After *P. viticola* inoculation, the expression of defense genes was generally weak in nontreated Pinot noir compared to nontreated Solaris in both 2016 (**Figures 6A, B**) and 2017 (**Figures 6C, D**), confirming the high basal defense level of the partially *P. viticola*-resistant Solaris. The control infected Solaris showed higher expression of genes responsive to SA, including *GST1*, *PR1*, and *PR2* in 2016 (**Figures 6A, B**) and *GST1*, *PR1*, and *HSR* in 2017 (**Figures 6C, D**) compared to Pinot noir. The expression of *PAL* was also higher in Solaris than in Pinot noir, suggesting that the basal resistance of Solaris to *P. viticola* can be linked to activated SA and phenylpropanoid defense pathways. However, after *P. viticola* inoculation, ISR-expressing plants by PTA-CT2 were primed for augmented expression of *PR1* in Pinot noir and *PR1* and *PR2* in Solaris in year 2016 (**Figure 6A**), while the transcript level of G*ST1*, *PAL*, and *STS* remained high in Solaris and comparable to control in 2016 (**Figure 6A**). The expression level of JA- and ET-responsive genes (*LOX9* and *ACO*) as well as that of *HSR* remained low in both cultivars and unchanged in the ISR-expressing plants compared to untreated plants. This highlights the prominent role of the SA signaling and phenylpropanoid pathways in the bacteria-mediated ISR against *P. viticola*.

Experiments performed in 2017 showed similar profiles with amplified gene expression in noninduced and ISR-expressing plants (**Figure 6B**). This demonstrates the ability of plants to respond and resist better after a second treatment in 2017. SA-related defense responses (*GST1*, *PR1*, and *PR2*) were much more expressed after *P. viticola* challenge. Interestingly, SA-responsive, but not JA- or ET-responsive, genes were activated in both cultivars after *P. viticola* challenge. Bacterium-mediated ISR against *P. viticola* is also accompanied by an impressive upregulation of *HSR* gene in Solaris, but not in Pinot noir. This result emphasizes the important role of the SA pathway in mediating ISR in both cultivars and further indicates that the HR, which is effective and strongly primed in Solaris, is deficient in the highly susceptible cultivar to *P. viticola*.

We also monitored transcript levels of the same genes after infection with the necrotroph *B. cinerea*. We showed that PTA-CT2 primed ET-responsive (*ACO*), but not JA- (*LOX9*) or SA-responsive genes (*GST1*, *PR1*, and *PR2*) in Pinot noir over the two consecutive years (**Figures 6C, D**). However, JA- and SA-responsive genes were highly upregulated in Solaris. PTA-CT2 treatment resulted also in a strong downregulation of *HSR*, a marker of cell death, in both cultivars after *B. cinerea* challenge (**Figures 6C, D**). These results suggest that the primed machinery of hypersensitive cell death by the beneficial bacterium may play a crucial role in strengthening resistance of Solaris against the biotrophic *P. viticola*, while induced resistance against the necrotrophic *B. cinerea* involves a decreased expression of *HSR* in both cultivars.

We also analyzed genes of the phenylpropanoid pathway *PAL* and *STS* that encode PAL and stilbene synthase involved in the biosynthesis of stilbenic phytoalexins. Both genes were significantly primed by PTA-CT2 in both cultivars upon *B. cinerea* inoculation, especially in 2016 (**Figure 6C**). The primed expression of PAL and STS was higher in Solaris than in Pinot noir. However, in both cultivars, the transcript level of PAL and STS remained low after renewing treatment with PTA-CT2 in 2017.

### PTA-CT2 Primes Phytoalexin Accumulation After Pathogen Inoculation in Both Pinot Noir and Solaris

**Figure 7:**
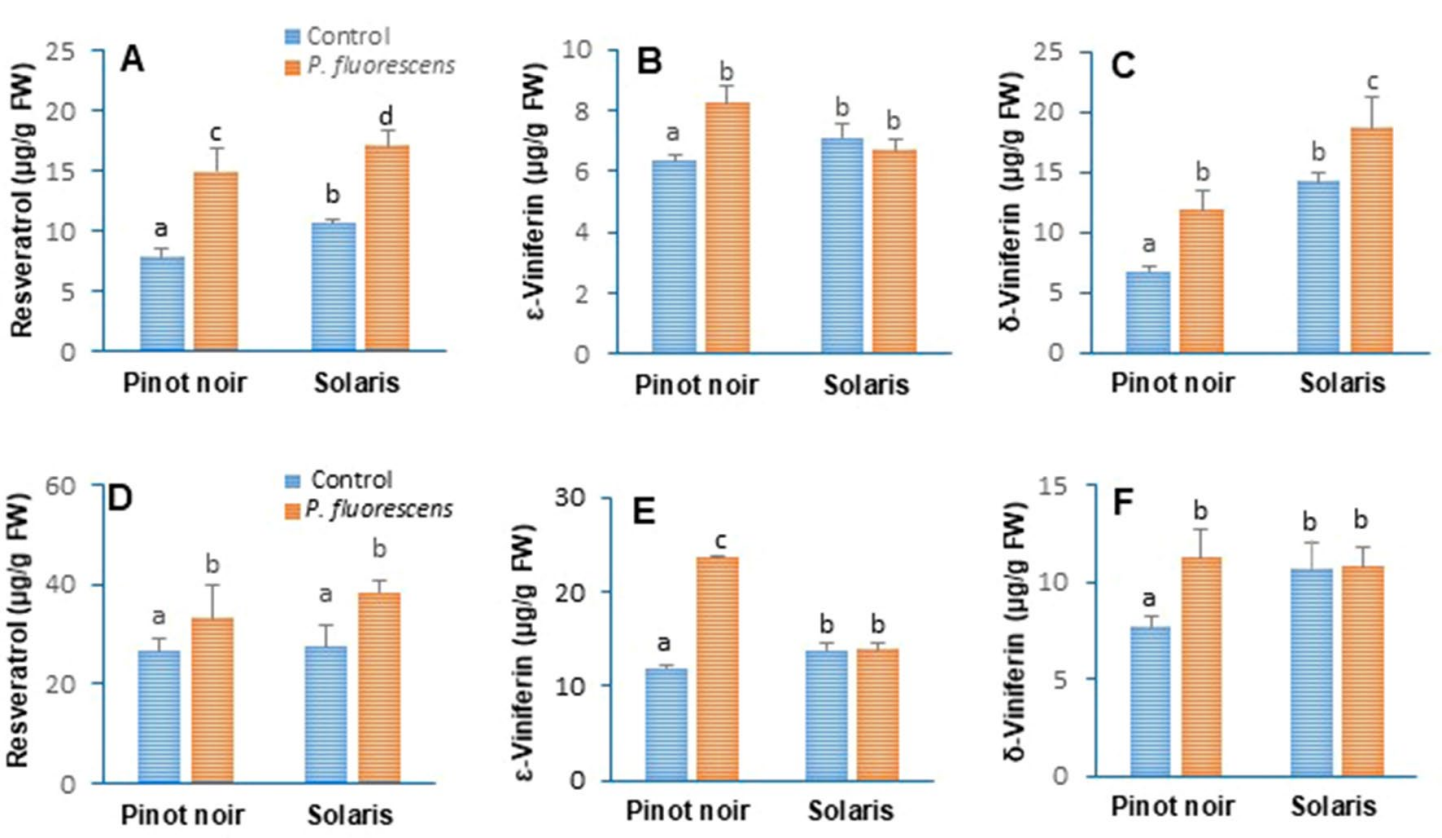
*Pseudomonas fluorescens* PTA-CT2 primes phytoalexin accumulation in leaf disks of Pinot noir and Solaris after *Plasmopara viticola* and *Botrytis cinerea* inoculation. Grafted plants were treated with *P. fluorescens* PTA-CT2 at the root level. Two weeks later, leaf disks were excised from control and treated plants and infected with 20 µl of 10^5^ spores per milliliter of *P. viticola* **(A–C)** or 5 µl of 10^6^ conidia per milliliter of *B. cinerea* **(D–F)**. The amounts of resveratrol **(A, D)**, *ε*-viniferin **(B, E)**, and *δ*-viniferin **(C, F)** were determined by ultra-performance liquid chromatography (UPLC) at 2 days post inoculation. Values corresponding to uninfected control or PTA-CT2-treated plants are similar to those before inoculation (**Figure 1**), with less than 1 μg g^−1^ FW. Data are the means from three independent experiments performed in 2016, and vertical bars indicate standard deviation. Different letters indicate statistically significant differences between the treatments (ANOVA Tukey test, P < 0.05).

Stilbenoid compounds are important secondary metabolites of grapevine that represent central phytoalexins and therefore constitute an important element of basal immunity. In this study, the difference between induced r esistance phenotypes of both cultivars was sought with respect to their output of stilbene accumulation. Results revealed significant differences in resveratrol, ε-viniferin, and δ-viniferin content between nontreated cultivars after pathogen inoculation (**Figure 7**). After *P. viticola* challenge, Solaris accumulated comparable amounts of resveratrol as Pinot noir did (**Figure 7A**), while the amounts of *ε*-viniferin (**Figure 7B**) and δ-viniferin (**Figure 7C**) were higher in Solaris than in Pinot noir. PTA-CT2 primed both *ε-* and *δ-*viniferin accumulation in ISR-expressing Pinot noir, while primed accumulation of resveratrol and δ-viniferin was recorded in Solaris in the 2016 season (**Figures 7B, D**). The primed δ-viniferin amount reached a higher level in challenged Solaris compared to the mock-infected plants and to Pinot expressing ISR (**Figure 7C**). Similar results were obtained over the 2017 year (data not shown), and no additional accumulation of these stilbenes was observed after *P. viticola* challenge. These results indicate that PTA-CT2-mediated ISR against *P. viticola* is tightly associated with priming stilbenoid compounds in the leaf tissues of both varieties.

Significant differences regarding the level of *ε*- and *δ*-viniferin were also recorded among cultivars after 48 h of *B. cinerea* infection (**Figures 7E, F**). Solaris exhibited a higher level of these stilbenoids than did Pinot noir, while both cultivars accumulated similar amounts of resveratrol (**Figure 7D**). PTA-CT2 treatment increased significant accumulation of resveratrol in both Pinot noir and Solaris after *B. cinerea* infection (**Figure 7D**). The beneficial bacterium also potentiated Pinot noir for enhanced accumulation of ε-viniferin (**Figure 7E**) and δ-viniferin (**Figure 7F**). No enhanced accumulation of these stilbenes was observed in Solaris, but their amount was maintained to a high extent in PTA-CT2-treated plants.

### PTA-CT2 Primes Differential Accumulation of Phytohormones in Grapevine Cultivars Depending on the Pathogen Lifestyle

**Figure 8:**
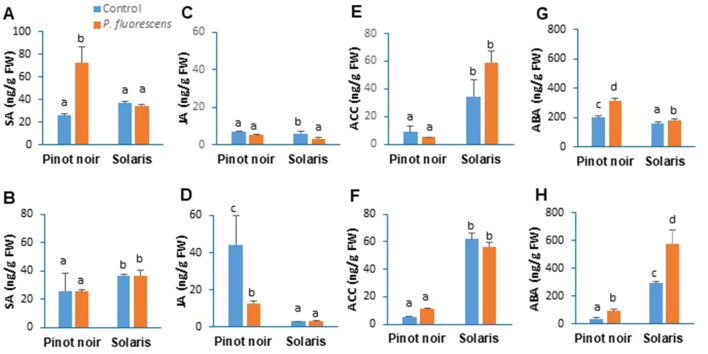
*Pseudomonas fluorescens* PTA-CT2 primes phytohormone production in leaf disks of Pinot noir and Solaris after *Plasmopara viticola* and *Botrytis cinerea* inoculation. Grafted plants were treated with *P. fluorescens* PTA-CT2 at the root level. Two weeks later, leaf disks were excised from control and treated plants and infected with 20 µl of 10^5^ spores per milliliter of *P. viticola* **(A, C, E, G)** or 5 µl of 10^6^ conidia per milliliter of *B. cinerea* **(B, D, F, H)**. The amounts of salicylic acid **(A, B)**, jasmonic acid **(C, D)**, 1-aminocyclopropane-1-carboxylic acid **(E, F)**, and abscisic acid **(G, H)** were determined by liquid chromatography (LC)–tandem mass spectrometry (MS/MS) at 2 days post inoculation. Values corresponding to uninfected control or PTA-CT2-treated plants are similar to those before inoculation (**Figure 2**). Data are the means from three independent experiments performed in 2017, and vertical bars indicate standard deviation. Different letters indicate statistically significant differences between the treatments (ANOVA Tukey test, *P* < 0.05).

To gain further insight into the link between resistance phenotypes primed by PTA-CT2 and hormonal signaling, we monitored the amount of SA, JA, ACC (a precursor of ET), and ABA in Pinot noir and Solaris leaf disks after infection with *P. viticola* and *B. cinerea*. Results from the 2017 season (**Figure 8**) showed differential phytohormone accumulation between cultivars after pathogen challenge. After *P. viticola* inoculation, SA was accumulated to similar levels in both cultivars (**Figure 8A**), while JA (**Figure 8B**) and ACC (**Figure 8C**) levels were higher in Solaris than in Pinot noir. The control infected Pinot noir showed a higher level of ABA compared to Solaris (**Figure 8D**).

Pretreatment with PTA-CT2 induced significant accumulation of SA and ABA by 3- and 1.7-fold, respectively, compared to the infected control (**Figures 8A, D**). The increased accumulation of SA is consistent with the upregulation of SA-responsive genes *GST1* and *PR2* in the susceptible variety after *P. viticola* challenge (**Figure 6B**). This highlights the important role of SA level and signaling in ISR triggered by *P. fluorescens* against *P. viticola*. In PTA-CT2-treated Solaris, a nonconsistent change was observed in SA (**Figure 8A**) and ACC (**Figure 8C**) content, which remained high after pathogen infection. However, a slight but significant accumulation of ABA was potentiated in the partially resistant Solaris (**Figure 8D**).

After *B. cinerea* infection, nontreated Solaris accumulated higher amounts of SA (**Figure 8E**), ACC (**Figure 8G**), and ABA (**Figure 8H**) compared to nontreated Pinot noir, which showed a greater accumulation of JA (**Figure 8F**). However, in PTA-CT2-treated Pinot noir, the amount of SA and ACC did not change, and only ABA accumulated after *B. cinerea* infection. Surprisingly, the JA content decreased after *B. cinerea* infection as compared to the infected control (**Figure 8F**). No additional accumulation of SA, JA, and ACC was observed in Solaris after infection. However, PTA-CT2 potentiated a strong accumulation of ABA in this cultivar after *B. cinerea* challenge.

## Discussion

In this study, we examined the immune responses triggered by *P. fluorescens* PTA-CT2 against biotrophic oomycete *P. viticola* and the necrotrophic fungus *B. cinerea* and compared these responses in two grafted grapevine cultivars with contrasting susceptibility to downy mildew, Pinot noir and Solaris, the former being susceptible and the latter partially resistant to infection. We provide evidence that *P. fluorescens* PTA-CT2 induces ISR against *P. viticola* and *B. cinerea* under greenhouse conditions by priming common and distinct defensive pathways depending on the basal immunity of genotype.

### PTA-CT2 Does Not Affect Basal Defenses but Elicits Differential Production of Phytohormones and Photosynthetic Efficiency Before Pathogen Inoculation

Our study indicates that grapevine cultivars displayed contrasting levels of basal defenses and phytohormones. As reported for other cultivars (Figueiredo et al., 2012), some SA-related genes were preferentially expressed in Solaris as partially resistant to downy mildew. Similarly, higher amounts of SA and ACC (ET precursor) were recorded in healthy leaves of Solaris compared to Pinot noir, which produces more JA. This contrast could support the hypothesis that the resistance conferred by *Rpv10* or *Rpv3* in Solaris (Schwander et al., 2012; Vezzulli et al., 2019) could be linked to the constitutive expression of some defense genes and specific hormonal balance in favor of SA production. However, PTA-CT2 did not induce any consistent change of basal expression of defense genes or the amount of phytoalexins in either cultivar. PTA-CT2 did not impact either SA or ACC levels but elicited differential accumulation of JA and ABA in leaf tissues. It is therefore suggested that PTA-CT2 may confer a hormonal homeostasis for each cultivar and thus prime plants for enhanced resistance upon subsequent pathogen attack. Our results also revealed some differences with the study performed with Chardonnay vitroplantlets (Gruau et al., 2015), which showed activation of systemic defense responses including expression of transcription factors and *PR* genes, as well as the synthesis of stilbenic phytoalexins in response to PTA-CT2. This difference could probably be due to the intrinsic difference of the plant models or grapevine varieties. Furthermore, the mode of action of PTA-CT2 seems to be different to that of other inducers of grapevine resistance against *P. viticola*, such as *T. harzianum* T39 (Perazzolli et al., 2012), thiamine (Boubakri et al., 2012), laminarin (Aziz et al., 2003), and its sulfated form PS3 (Gauthier et al., 2014), that directly elicit some defense responses even before infection.

Clear differences were also recorded between cultivars with respect to photosynthetic efficiency as indicated by higher values of Fv/Fm, ΦPSII, and ETR rate in Solaris than in Pinot noir. PTA-CT2-treated plants showed a significant increase of photosynthetic efficiency in both cultivars, highlighting an improvement of energy transfer within PSII after root treatment with PTA-CT2. This effect appears to be a common aspect of plant growth-promoting rhizobacteria (PGPR)-mediated defenses (Zhang et al., 2008; Miotto-Vilanova et al., 2016), suggesting that PTA-CT2 may initiate plant immunity by regulating the photosynthetic machinery as an energy supplier. Furthermore, enhancement of electron transfer is well known to reduce susceptibility to necrotrophic pathogens (Kangasjarvi et al., 2012). Altogether, the apparent increase of JA and ABA content and photosynthetic performance suggests that PTA-CT2 may mobilize the central metabolism of host plants, which could be crucial for the outcome of plant immunity. Further investigations are clearly needed to identify bacterial traits that govern the photosynthesis efficiency in grapevine plants.

### PTA-CT2 Induces ISR Against *P. viticola* and *B. cinerea* to Different Extents in Susceptible and Resistant Cultivars

As expected, Pinot noir and Solaris displayed contrasting phenotypic susceptibility to both *P. viticola* and *B. cinerea*. Over the two consecutive years, Pinot noir showed higher susceptibility to *P. viticola*, but it was more tolerant to *B. cinerea* compared to Solaris. These phenotypic differences could be attributed to contrasting pathogen infection strategies and host defenses against biotrophic and necrotrophic pathogens (Armijo et al., 2016). We also clearly demonstrated that PTA-CT2 strongly reduced downy mildew disease in both susceptible and partially resistant cultivars. To our knowledge, this is the first report characterizing effective protection of bacteria against *P. viticola* in* V. vinifera* genotypes. These effects are comparable to those induced by BABA in Chasselas (susceptible) and Solaris (tolerant) cultivars (Slaughter et al., 2008). PTA-CT2-induced resistance against gray mold disease was also confirmed in both Pinot noir and Solaris, consistent with our previous results using the Chardonnay cultivar (Verhagen et al., 2011; Gruau et al., 2015; Aziz et al., 2016). However, the level of induced protection was comparable between cultivars against *P. viticola*, while the efficiency of PTA-CT2 was greater in Pinot noir compared to Solaris. In both cases, the bacterium did not exert any direct effect on the pathogen growth or sporulation. Furthermore, it has been shown that PTA-CT2 can colonize grapevine roots, but not shoots (Gruau et al., 2015), suggesting that the protection mediated by this bacterium against both necrotroph and biotroph pathogens resulted from an ISR in grapevine plants. Although additional proof is needed, the results raise the possibility that the efficacy of bacteria may rely on its root colonization capacity that could be influenced by basal defenses of genotype.

### PTA-CT2 Primes Common and Distinct Defensive Pathways Against *P. viticola* and *B. cinerea* in Susceptible and Partially Resistant Cultivars

Our results clearly revealed an induced host priming state by *P. fluorescens*, with differential responsiveness between cultivars. After *P. viticola* inoculation, most responsive genes to SA (*GST1*, *PR1*, and *PR2*), *HSR*, and *PAL* were greatly upregulated, along with higher accumulation of JA and ACC in Solaris than in Pinot noir. Both cultivars accumulated the same amount of SA, but ABA content was higher in Pinot noir. This result can explain the contrasting phenotypes and supports the idea that the basal resistance of Solaris to *P. viticola* can be linked to an activated SA pathway, phytoalexin synthesis, and HR-like cell death, which are considered as key components differentiating genotypic resistance (Zyprian et al., 2016; Figueiredo et al., 2017; Vezzulli et al., 2019). According to our results, it has also been reported (Polesani et al., 2010; Malacarne et al., 2011) that *P. viticola* infection resulted in an induction of genes involved in ET biosynthesis more importantly in resistant than in susceptible cultivars. An important role of JA signaling in grapevine resistance against *P. viticola* has also been suggested in a study (Figueiredo et al., 2015) performed on two genotypes with different degrees of resistance to this pathogen. Similarly, the JA signaling pathway was shown to be implicated in induced resistance against powdery and downy mildews in grapevine by different elicitors (Hamiduzzaman et al., 2005; Trouvelot et al., 2008).

Interestingly, PTA-CT2 potentiated a high expression of SA-related genes and phytoalexin synthesis, while JA- and ET-responsive genes remained unchanged after *P. viticola* challenge. The primed responses seem to be enhanced by the second bacterial treatment in both cultivars, along with a strong expression of the HR-related gene in Solaris, indicating a common functional role of the SA signaling and stilbene accumulation in the PTA-CT2-induced resistance in both genotypes and more specifically of primed HR-like cell death in Solaris upon *P. viticola* inoculation. These results are at least partially in accordance with those reported with sulfated laminarin PS3 that primes SA- and HR-dependent defenses in both susceptible and tolerant cultivars (Trouvelot et al., 2008; Gauthier et al., 2014), but not with *T. harzianum* T39, which rather primes JA and ET pathways (Perazzolli et al., 2008; Palmieri et al., 2012) upon downy mildew infection. It has been shown that BABA-improved resistance of Solaris was accompanied by upregulation of genes involved in phytoalexin pathways (Slaughter et al., 2008). The upregulation of SA-related genes is consistent with the primed SA level, accompanied by ABA accumulation in the susceptible variety after *P. viticola* challenge. This suggests that ISR against *P. viticola* in the susceptible cultivar may be dependent on the coordinated action of both SA and ABA, rather than on HR-like cell death. The primed accumulation of ABA can be linked to its role in regulating stomatal closure (Ton et al., 2005) or callose biosynthesis and deposition (Flors et al., 2005), thus inhibiting pathogen spread and reducing disease infection.

However, after *B. cinerea* inoculation, Solaris plants showed enhanced expression of SA-related genes and accumulated higher amounts of SA, while Pinot noir rather accumulated JA. This can explain the contrasting susceptibility of Pinot noir and Solaris to the biotroph *P. viticola* and the necrotroph *B. cinerea*, respectively, as it is generally reported that JA and SA signaling cross-talk and have a mutual antagonistic effect on each other (Pieterse et al., 2009). Interestingly, PTA-CT2 primed ET gene expression and a high amount of JA in Pinot noir, while it potentiated high expression of JA- and SA-responsive genes and an increased level of ABA. This indicates that bacterium-mediated ISR to the necrotroph *B. cinerea* is dependent on JA/ET signaling and also on the basal immunity of each cultivar. The role of ABA in ISR needs further proof. PTA-CT2 treatment resulted also in an upregulation of *PAL* and *STS* genes and primed accumulation of resveratrol and viniferin in Pinot noir, whereas the amount of these stilbenes was maintained to a high extent in Solaris. These results confirm the prominent role of stilbenoids in strengthening resistance in grapevine plants against both biotrophic (Vezzulli et al., 2019) and necrotrophic pathogens (Gruau et al., 2015; Aziz et al., 2016). However, PTA-CT2 strongly reduced the expression of *HSR* in both cultivars after *B. cinerea* inoculation, suggesting that the primed machinery of hypersensitive cell death by the beneficial bacterium may play a crucial role in improving resistance of Solaris against the biotrophic *P. viticola*, while ISR against the necrotrophic *B. cinerea* seems to be associated with decreased HR-like cell death in both cultivars. This could be related to the necrotrophic nature of *B. cinerea*, which tends to trigger HR as a mechanism to provide the nutrient supply necessary for the infection process (Govrin and Levine, 2000).

**Figure 9:**
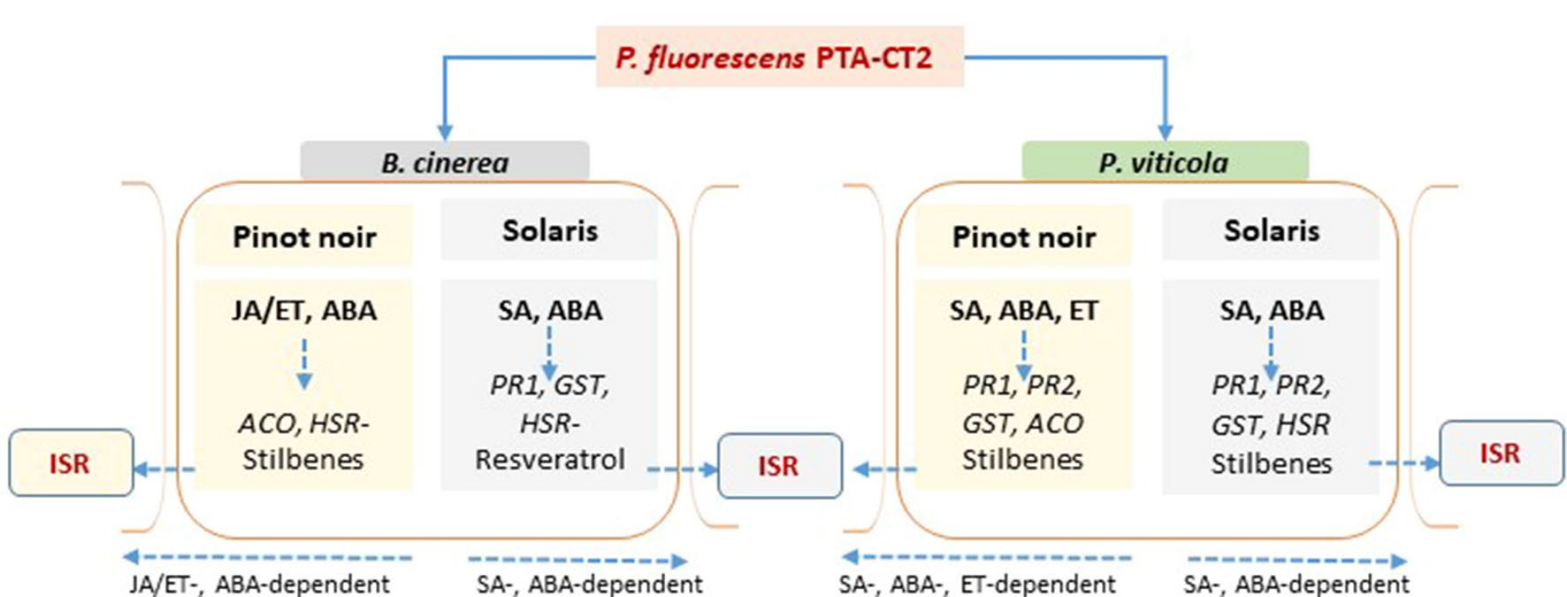
Proposed model summarizing defensive pathways primed in *Pseudomonas fluorescens*-induced systemic resistance against *Botrytis cinerea* and *Plasmopara viticola* in the susceptible Pinot noir and the partially resistant Solaris grapevine genotypes. The phytohormones are in bold, the genes are in italic, and the sign (−) in front of the *HSR* gene means downregulated expression.

Overall, we showed that grapevine cultivars displayed contrasting levels of basal defenses and phytohormones, as well as phenotypic susceptibility to *P. viticola* and *B. cinerea*. SA-related genes and accumulation of SA and ACC were preferentially expressed in the partially resistant Solaris, compared to the susceptible Pinot noir that required more JA signaling. The resistant cultivar also exhibited higher photosynthetic efficiency than the susceptible one. We also demonstrated that *P. fluorescens* PTA-CT2 induces ISR against *P. viticola* and *B. cinerea* by priming common and distinct defensive pathways depending on the basal immunity of genotype (**Figure 9**). Although PTA-CT2 seems to mobilize the host metabolism before infection, our results highlight an important role for SA-dependent defense and phytoalexin synthesis, but not JA and ET signaling in ISR against *P. viticola* in both susceptible and partially resistant cultivars. The priming state relies further on the strong expression of HR-like cell death in Solaris, while in the susceptible cultivar, it involves accumulation of ABA, rather than HR-like cell death. Interestingly, bacterium-mediated ISR against *B. cinerea* is dependent on JA/ET signaling and also on the accumulation of stilbenoids and weakening of hypersensitive cell death in both cultivars.

## Data Availability

All datasets generated for this study are included in the manuscript and the **Supplementary Files**.

## Author Contributions

AA, CC, and SL conceived and designed experiments; SL, FR, AS, and PT-A performed experiments; FR, AS, EN-O, CC, and AA gave their expertise for all steps of this work; AA, SL, FR, AS, and EN-O analyzed and discussed experiments; AA and SL wrote the paper with the contribution of all co-authors. All authors read and approved the manuscript.

## Conflict of Interest Statement

The authors declare that the research was conducted in the absence of any commercial or financial relationships that could be construed as a potential conflict of interest.
