# Supplementary figures and images for: Strengthening Grapevine Resistance by Pseudomonas fluorescens PTA-CT2 Relies on Distinct Defense Pathways in Susceptible and Partially Resistant Genotypes to Downy Mildew and Gray Mold Diseases
Source: Front Plant Sci. 2019 Sep 18;10:1112. doi: 10.3389/fpls.2019.01112 (PMC6759587; doi:10.3389/fpls.2019.01112)

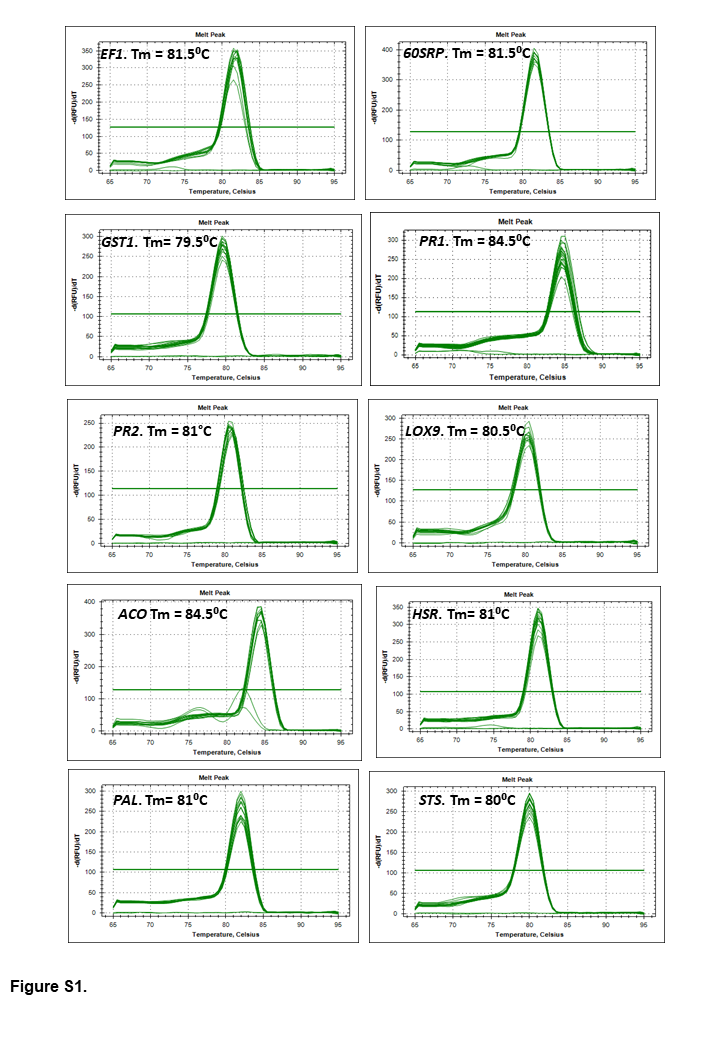

Supplement: Supplementary file 1 [file Image_1.tif]

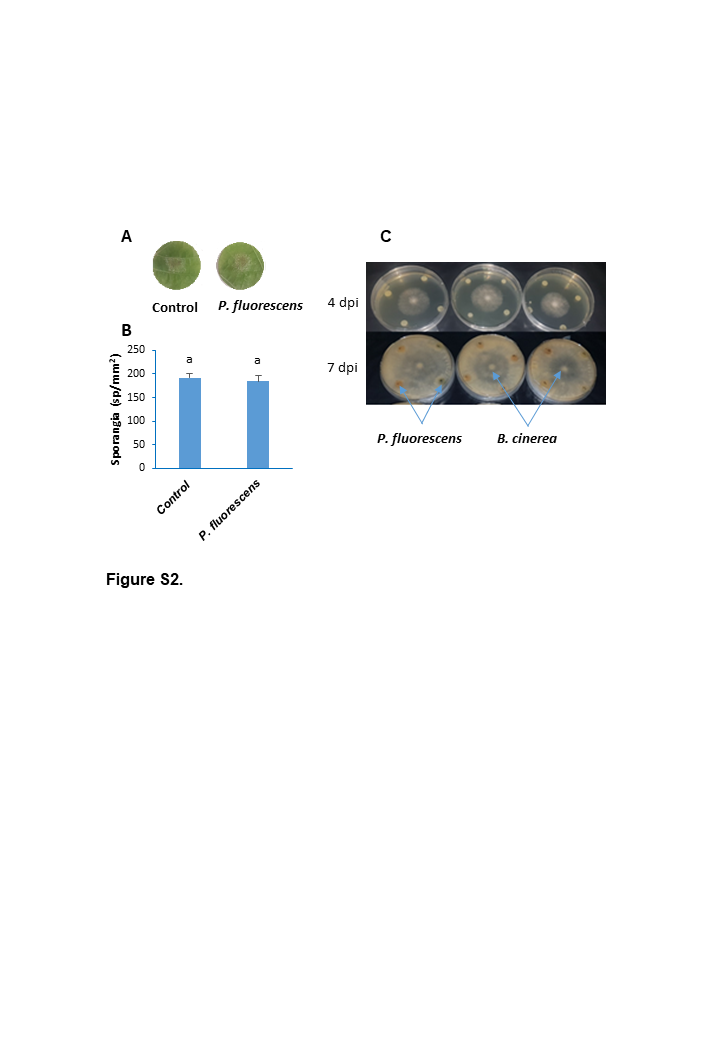

Supplement: Supplementary file 2 [file Image_2.tif]
