# Supplementary material for: Strengthening Grapevine Resistance by Pseudomonas fluorescens PTA-CT2 Relies on Distinct Defense Pathways in Susceptible and Partially Resistant Genotypes to Downy Mildew and Gray Mold Diseases
Source: Front Plant Sci. 2019 Sep 18;10:1112. doi: 10.3389/fpls.2019.01112 (PMC6759587; doi:10.3389/fpls.2019.01112)
Supplement: Supplementary file 3 [file Table_1.docx]

Table 1 Primer sequences used for RT-PCR analysis of defense-related genes

| **Gene** | **Accession no.^1^** | **Forward Primer (5’-3’)** | **Reverse primer (5’-3’)** |
| --- | --- | --- | --- |
| *HSR* | NM_001281193.1 | GGGACAGTGCTGGAGGAAAC | ATCCAGTCGGTTAATGCCAAA |
| *GST1* | NM_001281248.1 | TGCATGGAGGAGGAGTTCGT | CAAGGCTATATCCCCATTTTCTTC |
| *PAL* | XM_003635637.1 | TCCTCCCGGAAAACAGCTG | TCCTCCAAATGCCTCAAATCA |
| *STS* | NM_001281117.1 | AGGAAGCAGCATTGAAGGCTC | TGCACCAGGCATTTCTACACC |
| *LOX9* | NM_001281249.1; XM_002280615.1 | CCCTTCTTGGCATCTCCCTTA | TGTTGTGTCCAGGGTCCATTC |
| *PR-1* | XM_002273752.2 | GGAGTCCATTAGCACTCCTTTG | CATAATTCTGGGCGTAGGCAG |
| *PR-2* | NM_001280967.1 | TCAATGGCTGCAATGGTGC | CGGTCGATGTTGCGAGATTTA |
| *ACO* | XM_002273394.3 | AAGGTCAGCAACTACCCTCC | CGCATCGGTGGAACATCAAT |
| *EF1α* | XM_002284888.1 | AACCAAAATATCCGGAGTAAAAGA | GAACTGGGTGCTTGATAGGC |
| *60RSP* | XM_002270599.1 | ATCTACCTCAAGCTCCTAGTC | CAATCTTGTCCTCCTTTCCT |

^1^ NCBI accession number
